# How Virtual Reality Technology Has Changed Our Lives: An Overview of the Current and Potential Applications and Limitations

**DOI:** 10.3390/ijerph191811278

**Published:** 2022-09-08

**Authors:** Ayah Hamad, Bochen Jia

**Affiliations:** College of Engineering and Computer Science, University of Michigan-Dearborn, Dearborn, MI 48128, USA

**Keywords:** virtual reality (VR), simulation, immersive, non-immersive, application, education, training, games, medical, healthcare, entertainment

## Abstract

Despite virtual reality (VR) being initially marketed toward gaming, there are many potential and existing VR applications in various sectors and fields, including education, training, simulations, and even in exercise and healthcare. Unfortunately, there is still a lack of general understanding of the strengths and limitations of VR as a technology in various application domains. Therefore, the aim of this literature review is to contribute to the library of literature concerning VR technology, its applications in everyday use, and some of its existing drawbacks. Key VR applications were discussed in terms of how they are currently utilized or can be utilized in the future, spanning fields such as medicine, engineering, education, and entertainment. The main benefits of VR are expressed through the text, followed by a discussion of some of the main limitations of current VR technologies and how they can be mitigated or improved. Overall, this literature review shows how virtual reality technology has the potential to be a greatly beneficial tool in a multitude of applications and a wide variety of fields. VR as a technology is still in its early stages, but more people are becoming interested in it and are optimistic about seeing what kind of changes VR can make in their everyday lives. With how rapidly modern society has adapted to personal computers and smartphones, VR has the opportunity to become the next big technological turning point that will eventually become commonplace in most households.

## 1. Introduction

This literature review aims to contribute to the library of literature on the applications of virtual reality (VR), how they are currently used and can be used in the future, and some of the strengths and difficulties that come with using VR.

Virtual reality (VR) refers to a computer-generated, three-dimensional virtual environment that users can interact with, typically accessed via a computer that is capable of projecting 3D information via a display, which can be isolated screens or a wearable display, e.g., a head-mounted display (HMD), along with user identification sensors [1]. VR can mainly be divided into two categories: non-immersive, and immersive [2]. Non-immersive VR utilizes a combination of screens surrounding the user to present virtual information [3]. A typical example of this is driving or flight simulations in which the user sits in a chair with multiple screens around them, giving them the feeling of being in the cockpit or driver’s seat without being fully immersed. Immersive VR refers to using a wearable display, e.g., HMD, to track a user’s movement and present the VR information based on the position of users [4], which allows them to experience 360 degrees of the virtual environment. This immersive experience is what most people think of when it comes to VR and is one of the most marketable aspects of VR technology. In between immersive and non-immersive VR, there is also augmented reality (AR). AR makes use of computer-generated imagery that is overlayed on physical elements in the real world, which can be found in many applications, such as stores providing a virtual fitting application for people to “try on” clothes. Mixed reality (XR) represents the spectrum between the physical and digital worlds, combining AR and VR to allow users to both immerse themselves in a virtual world while also being somewhat grounded in reality.

The concept of VR was first introduced in the 1960s, with Morton’s creation of the Telesphere Mask and the Sensorama [5]. The original technologies served the purpose of immersing the user in the video display around them, making them feel like they are a part of the video. The Ultimate display was an idea developed by Ivan Sutherland [6], operating on a similar concept of allowing the user to feel immersed in a computer-generated environment using multiple input and output devices [7,8]. Following the creation of the Sensorama and the idea of the Ultimate display in the 1960s, the next large boom in VR technology development occurred in the early 2010s. During this period of time, VR was still considered a gimmick—it was expensive and was not considered a technology that would ever become popular with the general public. This, however, started to shift in 2012, when Palmer Luckey debuted his prototype for the first Oculus [9]. In 2014, Facebook acquired Oculus after seeing the interest it garnered, leading to a significant increase in the popularity of VR devices for home use. Since then, VR has grown to become more popular and accessible to the everyday consumer, with more VR headsets available on the market, such as the HTC Vive, Samsung VR, Oculus, Google Cardboard, and more.

Despite VR being initially marketed toward gaming, there are many potential and existing VR applications in various sectors and fields, including education, training, simulations, and even in exercise and healthcare. Unfortunately, there is still a lack of general understanding of the strengths and limitations of VR as a technology in various application domains. Some of the largest issues with current VR technology are hard to overcome and can span from technical to financial and health issues. Technological limitations regarding users feeling uncomfortable or ill while using a VR headset, the inaccessibility of this technology to most people due to the high price of the associated hardware, and the lack of technical standardization are all current issues that the tech industry is hoping to overcome with research and future improvements.

Overall, this literature review serves the purpose of covering how different types of VR applications can be utilized, as well as providing information on the advantages and drawbacks of using VR technology in various application domains.

## 2. Methods

In order to present a reliable literature review, an extensive search was performed using common journal search engines/websites, e.g., Google Scholar, JSTOR, MDPI, ResearchGate, PubMed, and Science Direct, which includes peer-reviewed studies and articles. Keywords and phrases used in searching for sources include a combination of “VR” or “virtual reality” with “Education”, “Simulation,” “Games”, “Virtual”, “Immersive”, “Non-immersive”, “Training”, “Application”, “Manufacturing”, “Industrial”, “Medical”, “Healthcare”, and “Entertainment”. The variety in keywords helped yield different results for VR not only as a technology but also in major use cases where it has already been utilized for different industries and fields. The gathered papers and articles were then reviewed to further select representative and up-to-date evidence.

**Figure 1:**
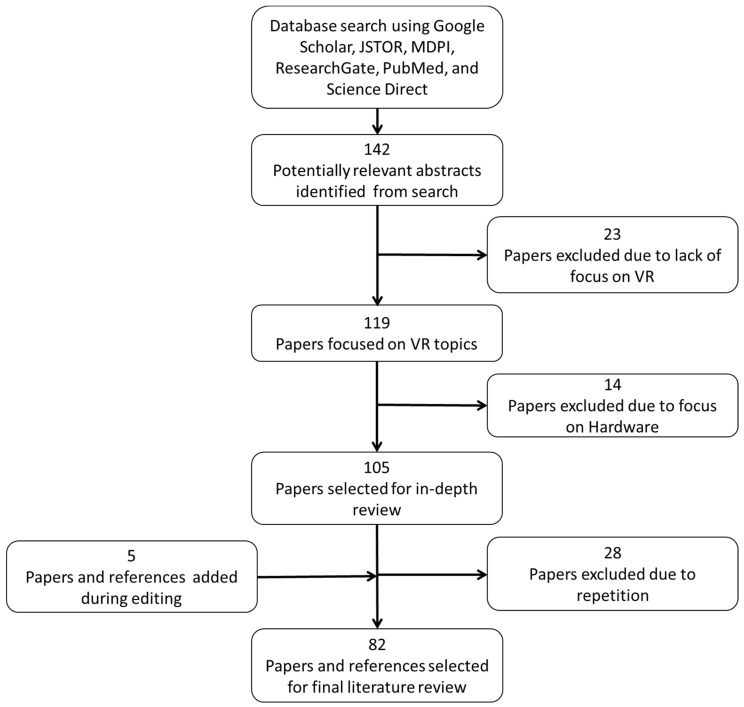
General structure of the paper selection and literature review.

Papers were selected with the goal of providing sufficient coverage of the topic by presenting an overarching summary rather than an exhaustive review of every type of application within VR. Having a large variety of papers does not guarantee that every particular use case of VR is covered, but it does provide a wide breadth of use cases of VR that are currently applied, as well as opportunity spaces for VR applications in the future. As shown in Figure 1, 145 papers were initially collected, but only 77 were thoroughly reviewed to provide enough coverage without unnecessary advanced technical details. Five additional papers and articles were added after review to accommodate additional information, resulting in a total of 82 sources used for the final literature review.

Included papers were those that clearly presented a specific VR application, those that showed clear negative or positive outcomes of VR usage, or papers that provided relevant background information on a specific VR technology. Exclusion criteria included disregarding papers that had an overt focus on VR hardware components, excluding studies that may have mentioned VR without it being the focus, and rejecting papers that became repetitive after utilizing other papers on similar topics. The following sections provide detailed reviews based on various VR applications and domains.

## 3. Reviews of VR Technology Applications

The technological applications of VR have advanced to a point where they can be applied to an extensive range of fields and industries outside of just gaming or entertainment. Many have started to take advantage of VR in performing tasks that are hard to practice due to limited resources or the inherent risks and dangers associated with said tasks that can sometimes lead to catastrophic consequences. The greatest strength of VR is that it opens up opportunities for people to practice these tasks in a safe capacity while also being immersed enough for it to feel realistic and transferable to the real world and depict almost any situation accurately [10]. This section covers some of the main categories of VR applications and provides examples of how these applications are applied or can be applied to different use cases across various fields.

One of the most widely used and largely applicable applications of VR is the simulation aspect, which can be uniquely created and customized to suit users’ needs. There are two main types of simulations: immersive and non-immersive. As mentioned above, non-immersive VR simulations usually include multiple screens and some type of platform or apparatus that mimics the activities or tasks in reality [3]. Immersive VR simulations differ in terms of using HMDs in place of screens and can either utilize a control platform or apparatus such as the ones used in non-immersive simulations [11] or can instead be fully contained within a virtual setup and require no external setups or platforms. Whether users opt for immersive or non-immersive VR simulations, there is no significant difference in the performance, and the results appear to be very similar in fulfilling the simulation’s purpose [12]. There is, however, a slight advantage to using immersive VR simulations with HMDs, as they are capable of fully immersing the user in the simulated environment and giving them a more thorough experience [13].

### 3.1. Industrial Simulation Applications

VR simulations have many applications that can span from training simulation to prototyping, designing, and testing tools and objects. Some commonly used VR simulations in the industrial domain include driving simulators, flight simulators for pilots, and combat simulators for military personnel, all of which provide training to users in highly dangerous circumstances without putting them at risk during the training process [14]. Among the many use cases, two typical simulation applications are further discussed in the following sections.

#### 3.1.1. Driving Simulations

One major use of VR simulations is driving simulations for both driving training and within the automotive industry; VR provides the ability to create driving simulations in which users can be placed in risky driving scenarios without real danger [15]. Driving simulators can be useful in multiple capacities, such as observing driving behavior to collect data or training inexperienced drivers in a low-stress environment.

VR driving simulations can be used to train young or novice drivers and help them understand their mistakes or point out some bad driving habits they need to adjust. Within a simulation, drivers can be placed in a virtual vehicle within an environment resembling a cityscape, with their behaviors and actions observed and recorded to later analyze for any issues or mistakes or to see if the drivers made the correct decisions in a given scenario [16]. After conducting the simulation, drivers can be informed of their mistakes and receive feedback about how to improve their behaviors in an actual driving situation. These driving simulations can also be beneficial in training young drivers with neurodevelopmental disorders such as autism spectrum disorder (ASD) [17], who may otherwise have difficulties learning in an uncontrolled environment.

Another application of VR driving simulations is the ability to collect real-time data on how users react to different scenarios as drivers on the road in a simulated environment. This data can be used in multiple capacities, such as designing better safety features in a vehicle, providing a better user experience for drivers, developing training modules for drivers, and for use in autonomous vehicle (AV) research and development. AVs have been an emerging field of technology that will continue to develop and advance, with VR simulations continuously providing opportunities for safe and efficient data collection and user testing [18]. One common issue in the field is developing trust between users and autonomous vehicles and understanding how to mitigate the distrust most people have in this technology [19]. It is important to ensure users have a certain level of trust in an AV so as to ensure drivers take over when appropriate. Accordingly, putting users in a VR driving simulation in which they interact with an autonomous vehicle virtually can yield substantial amounts of data on how users behave within that environment while also ensuring that users feel safe in the process and can become accustomed to being in an AV [20].

#### 3.1.2. Product Design and Prototyping

One application of VR that can be useful is the ability to look at 3D models in a virtual space in a way that is difficult to visualize via a screen. Prototypes or preliminary designs for products can be modeled and shown in a virtual environment for test and evaluation purposes [21]. One significant advantage of showing these models in VR is presenting a virtual prototype or part without spending a lot of time, money, effort, or material on building the prototype in real life. Through simulations, VR can also show how the product would react under different conditions. Simulations can be run in VR to show the effect of different interactions between the prototype and surrounding subjects [22]. This can help the prototype designers determine if any areas of the prototype need to be improved based on the simulated interaction results. The ability to see the product in a virtual environment can also provide the ability to make changes to VR design for a quick turnaround and faster results, which could increase the speed of prototyping, reduce prototype production waste, and increase the understanding of the functions of the prototype.

### 3.2. Education

Educational applications of VR have not been utilized much yet, but there are many promising examples and studies of how beneficial VR can be in an educational environment. Using VR can help increase student attention by keeping them engaged with what is happening inside the VR environment [23,24]. Most teenage students find it challenging to pay attention in class, especially when they feel that the discussed topics are not relevant to them. When students use exciting technologies such as VR, they are more interested and engaged with what they are learning while immersed in a virtual environment [25,26]. VR headsets are also useful in blocking out visual and auditory distractions, creating an opportunity for the student to focus on teaching materials better. Such VR approaches open up more opportunities for teachers to interact one-on-one with students and have more useful and beneficial teacher–student interactions [27].

VR also provides the opportunity for students to construct and practice their own knowledge by being able to engage in meaningful experiences. Students are able to immersively engage in educational activities and gain a better understanding of the topic at hand [28]. VR also has the capability of transporting students to different environments, allowing them to learn and explore various concepts safely and efficiently. This can be especially useful to demonstrate environments that are impossible to visit in reality, such as underwater or space [29,30].

Mixed reality can be considered an extended VR application, which can be applied to real learning environments, such as exploring laboratory experiments [31]. Students can wear an HMD that shows information and instructions about the laboratory they will experience and can interact with items in reality to recreate what is simulated to them in VR. Essentially, students are still fully aware of their surroundings while also having a better visual understanding and representation of their task, which can help reduce mistakes, allow students to be more independent, and keep students interested and engaged.

With the start of the COVID-19 pandemic, there has been a sudden increase in virtual learning, with many classes being held via online meeting platforms and others being fully asynchronous. VR offers a new, unique approach to asynchronous learning; VR can create a learning environment in which a student can participate in lectures and ask questions to virtual instructors with pre-generated answers [32]. It is particularly important for students to feel immersed in the virtual environment in order to keep them engaged [33]. Virtual environments can be created to look just like real-life classrooms where students can walk around and work with other students on assignments [34]. The issue with asynchronous classroom experiences is that not all of a student’s questions will necessarily be answered; information will be limited to what is currently updated within the virtual experience. Thus, VR-based virtual education does provide a better experience to students than watching videos online, but it cannot replace the experience of being in a classroom with teachers who can directly engage with students.

With VR technology further advancing, VR could also be used for live, synchronous classes where students can engage with classmates and teachers from the comfort of their homes in real time. This would have been especially beneficial when schools were closed due to the pandemic, but it can also provide a way for students to attend classes while experiencing health difficulties, traveling, or living in other countries, etc. Even though live classes have not yet really been held using VR, such applications can be developed in the future, especially with some of the current development being made in both asynchronous learning and social interaction.

### 3.3. Public Health

Another domain in which VR has been utilized is within public health and wellness. Due to the immersive nature of VR, it can be used to simulate experiences that can directly impact people’s health. Some examples include providing immersive training simulations to medical personnel, offering a new method of exercise or meditation, and presenting therapists with opportunities to better help and understand their patients.

#### 3.3.1. Medical Training

VR simulations provide the opportunity for medical professionals to practice procedures before operating on a patient, which has proven to help provide patients with better outcomes more consistently and reduce the incidence of mistakes. Preparation and practice in VR help improve patient outcomes because medical personnel are better prepared for each patient’s unique circumstances before operating [35,36].

In terms of learning how to perform procedures, medical students can train in an interactive virtual environment that can be programmed with different scenarios, which allows a student to experience real-life scenarios with virtual patients [37]. The virtual environment can be programmed in a multitude of diverse ways so the student can be prepared and better accustomed to different types of scenarios they may face with future patients. The simulation can be programmed so that a video can be played, showing how to effectively use a tool or object when the user looks at it [38]. The simulation can also provide hints or step-by-step instructions to students so they know how to perform the surgery properly. All these practices are much more hands-on than reading a textbook and more realistic than practicing on mannequins with minimal risks to a real patient, which makes VR a perfect tool to assist student learning.

Medical students are not the only ones who can benefit from VR simulations; seasoned medical professionals and surgeons can also benefit from this technology. Patient-specific virtual reality simulations (PSVR) are a technology that allows doctors to practice actual upcoming operations in VR [39]. This technology allows surgeons to practice customized procedures to match their patients’ specific needs and circumstances. A patient’s medical history and physical attributes can be created in the simulation and programmed with the most likely outcomes. When a surgeon performs a task or action in the simulation, the appropriate or most likely reaction can be programmed to simulate what would occur in real life under the same circumstance. This provides an opportunity for surgeons to plan out their surgery beforehand in a virtual environment, allowing them to be better prepared and more confident in their plan for the surgery ahead [40].

#### 3.3.2. Exergaming, Fitness and Sports

With the initial focus of VR being on gaming, developers saw an opportunity for the emergence of a genre of games called exergames, in which users participate in physical activities to achieve the goals of the game. “The core concept of exergaming rests on the idea of using vigorous body activity as the input for interacting with engaging digital game content with the hope of supplanting the sedentary activity that typifies traditional game interaction that relies on keyboards, gamepads, and joysticks” [41]. VR games tend to fall under the category of exergames by requiring the user to stand up and move around in order to interact with the environment. Games such as *Beat Saber* (Beat Games, Prague, Czech Republic) make the user move around frequently to fulfill the game’s requirements.

Using VR as a workout tool helps gamify exercise, which can greatly assist users in staying motivated and engaged by providing them with goals to achieve during their workout. A study performed by Segura-Orti on dialysis patients shows that patients that used VR exercises instead of conventional physical activities had an increased level of physical activity compared to those who worked out using conventional methods [42,43]. This is probably due to the more enjoyable experience of getting exercise in game form that real life has failed to achieve with exercise apps and challenges. Some current examples include the implementation of treadmills and stationary bicycles with VR applications that allow users to physically run/cycle in place while virtually traveling through a virtual environment. These types of immersive experiences can make users’ workouts more enjoyable and can help encourage those new to fitness to start exercising from home in a new and exciting fashion.

VR technology is also being utilized in sports, where it is used to train athletes to improve their skills and can help provide them with physical therapy and rehabilitation. In terms of athletic training, VR presents a great method of perceptual-cognitive skills training [44], where users are able to experience and learn from video-based playback in an immersive environment rather than on a screen. This can be especially useful in customizing training for players in large team sports, such as football, basketball, or soccer [45]. VR allows individuals to repeatedly practice skills with lower risks of harm, which helps reduce injury. When injuries do occur in the real world, VR can be used in the rehabilitation process by allowing athletes to train from anywhere and at any time, even in the absence of a trainer or facility.

#### 3.3.3. Therapy and Meditation

Another use of VR is in mental health therapy and meditation. The immersive nature of VR provides the flexibility to create various types of environments or experiences. Accordingly, VR can be used to experience situations that are hard to come by in real life, or that can be dangerous to go through in real life. For example, for those who suffer from post-traumatic stress disorder (PTSD), VR can be a way to experience situations that can trigger traumatic events within a safe, controlled capacity. Specific scenarios can be recreated in a virtual environment, and the patient can experience them in the presence of a therapist in order to receive help dealing with their trauma [46]. This type of therapy is similar to exposure therapy, in which patients confront what triggers them in order to slowly heal from their trauma [47].

For people who have certain disorders that may be hard to explain with words, VR can be a safe way to put people in scenarios that may trigger their disorders and observe their behaviors. Allowing a therapist to observe the situation can give them a better insight into why their patient is reacting in a certain way, which will allow them to better treat their patient [48].

Another application of VR is to use the immersive nature of the technology for meditation purposes. With the ability to experience a calm virtual environment that fully blocks distractions, VR presents a unique form of meditation that may be otherwise difficult to achieve at home. Studies on the use of VR in meditation have shown a slight increase in positive effects and a state of mindfulness in users after the meditation experience [49]. One study showed that VR meditation was more successful in reducing pre-exam anxiety in college students than watching a meditation video, where 71% of those using VR reported lower anxiety levels compared to 47% of the control group [50]. VR mediation has been shown to be useful in calming healthcare workers, especially during the COVID-19 pandemic. Virtual reality plus neurofeedback (VR + NF) meditation was shown to decrease the user’s anger, tension, depression, vigor, fatigue, and confusion [51]. Navarro-Haro et al. experienced an immersive VR mediation simulation and reported an increase in mindfulness and a reduction in negative emotional stress [52]. They were also less sad and less angry after the simulation. Mediation experts acknowledge that meditation with VR can be an immensely helpful and unique experience that is not yet fully utilized, and studies such as the one discussed here show promising results for this use of VR.

### 3.4. Social Interaction

VR provides the ability to transport users to a virtual environment in which they can interact with other users. This provides an opportunity to create social connections that may otherwise be hard to create or maintain. Social interaction via VR can be especially helpful for those with autism, as it provides a way for them to practice their communication skills. Users are able to participate in virtual cognition training to better improve their social skills, such as emotion recognition, social attribution, and analogical reasoning [53]. There are even programs in which young adults with high-functioning autism can participate that are designed with the purpose of increasing their social skills. These programs train users to better recognize facial expressions, body language, and emotions from a person’s voice [54]. These programs have lasting effects on the users, as they gain the ability to recognize other people’s emotions within the training that they can carry forward in their lives.

Social virtual reality also provides a new way for people to connect over long distances. Virtual spaces can be created in a VR environment and allow users to interact with each other in a realistic setting; users can have realistic avatars and talk to each other as if they were face-to-face [55]. This method of communication can be as effective as talking to another person in real life as long as the users feel immersed in the environment. When the users are immersed in the virtual environment, they have a better sense of presence, and their responses are more genuine [56]. This was especially popular during the COVID-19 pandemic when social distancing and travel restrictions made it much harder for people to see and speak with their loved ones [57]. Being able to attend events and experience activities with others via VR has provided a substitute for real-life interactions that is more realistic than merely speaking over the phone or via video chat [58].

### 3.5. Entertainment

The most prominent application of VR among the general public is within the sphere of entertainment, with VR offering new ways for users to experience several types of media in an immersive capacity.

One such form of media consumption within VR is watching movies, shows, or videos. VR offers new ways for users to experience visual media due to its ability to immerse users in a virtual world. VR displays are able to play 360° videos and allow the users to move around in the virtual environment, which provides the user with a more immersive experience and allows them to interact with the world as they see fit [59]. Users now have more control over what they want to pay attention to in a video and can experience videos in a whole new way. 

Another application is virtual travel and tourism. Virtual tourism allows users to experience immersive tourism in simulated environments based on real landscapes or locations. This can make travel attainable to many people that would otherwise not be able to afford the time or money needed to physically visit faraway destinations. Examples of VR tourism include virtual museum visits, navigating areas using applications such as Google Street View, and virtual tours of popular destinations such as the Grand Canyon or the Great Wall of China. The concept of virtually visiting other countries or worlds has existed since the 90s [60], but there was a boost in interest recently due to travel constraints during the COVID-19 pandemic [61], with more people seeking travel experiences from the confines of their homes.

Live music is another form of entertainment that seems to be gaining traction as another large application of VR. Virtual reality has the ability to change the way people experience concerts, offering users the ability to attend and enjoy concerts from anywhere in the world. Prerecorded concerts are already available as a VR experience, with videos of the concerts filmed in 360 using omnidirectional cameras, allowing users to move their heads around and feel like they are physically present at the concert [62]. This can be an opportunity for users who do not have the ability to travel or could not get tickets to still enjoy the show. This will also allow users to see parts of the concert they could not see even if they were there due to cameras either being positioned on stage or close to the stage. The livestreaming of concerts in VR is still not technologically applicable, but it seems like the music industry is aiming to make it a reality at some point in the future with further VR development. As part of the most significant applications of VR, gaming has gained huge popularity recently, with headsets becoming more accessible and game developers investing more in the VR landscape. Many users have purchased VR headsets to play popular games such as *Beat Saber*, *Super-Hot*, and *Job Simulator* (Menlo Park, Prague, Czech Republic), some of the top-selling VR games. Besides designated VR games, many other games that were not initially made for VR are also being developed to include this capability and expand the options gamers have concerning their in-game experience. The rise of VR gaming popularity in recent years owes to the immersive capabilities of HMDs to immerse the users in the game environment, blocking out all external distractions [63] and giving the users a better sense of presence [64]. Players can experience the game from their point of view, which allows users to experience games in a whole new way [65].

## 4. Limitations and Side Effects of VR

Despite VR being a powerful and versatile tool, current VR technology has some evident limitations and drawbacks. These limitations include technological limits on what VR can do, how accessible VR is to the general public, and some of the side effects of using VR devices.

### 4.1. Technological Limitations

As a technology still in the earlier stages of development on a grand scale, VR has made significant leaps in evolution. Still, more substantial progress must occur before VR can be fully utilized in all possible applications and purposes.

Right now, the standardization of VR technology and presentation is still limited [66]; every developer may have their own interface specifications and functionality associated with their technology, and applications are not easily transferable between devices. The only standardization that can be observed as of now tends to be with popular games that are developed to be used across different VR platforms. It is also hard to troubleshoot bugs and receive proper support for any issues due to the lack of standardization. Hopefully, with time and progress in VR development, the technology can become more streamlined and provide better usability for users and transferability between devices. There are currently efforts to standardize VR, but these efforts are new, and the process is still in its infancy [67].

Other issues include hardware and software requirements for professional VR development, as most VR development software tends to take up a lot of data space on computers and have high-power consumption [68]. VR headsets also tend to be very heavy and can cause physical strain on users, causing headaches and pain, especially around the neck and shoulders [69]. As of now, it is not yet known what kind of detrimental effects VR use will have on users’ eyesight, but it is known that it can cause strain, especially with prolonged usage [70].

Another common issue is the lag between the user’s movements and the visual display within a VR headset [71]. A lot of the time, the headset’s tracking does not keep up properly with the user’s movements, which not only decreases their immersion but can also cause dizziness or “cybersickness,” which is explained in more detail below [71,72].

#### Cybersickness

One of the crucial issues with VR usage is VR-induced motion sickness, or “cybersickness” [73,74]. Cybersickness is a phenomenon where users will feel symptoms similar to motion sickness (i.e., nausea, dizziness, lightheadedness) as a result of using a VR device [71]. It is not yet known exactly why this occurs, but there are a few theories to explain this phenomenon. The most likely theory is known as the “sensory conflict theory,” which states that the excessive mismatch between the motion a user perceives visually and the lack of the corresponding movement in their body causes a conflict [71,72,75]. This happens when there is a disparity between the user’s visual system and vestibular system, which is the sensory system responsible for providing the brain with information about motion, head position, and spatial orientation [76]. Another explanation for cybersickness is the “ecological hypothesis”, which states that when people are not able to perceive or react to new dynamic situations, postural instability occurs [77].

Cybersickness does not always come with virtual experiences, but the issue can be exacerbated by several factors. Some individual factors include prolonged VR exposure; the user’s predisposition to motion sickness, fatigue, or nausea; and how adapted a user is to VR applications [71,78]. Cybersickness symptoms also seem to be less frequent when users are sitting instead of standing. Symptoms tend to worsen when a user is experiencing a high-speed simulation or game. Being a passive participant makes users more susceptible to symptoms than when they are in control of the simulation [71,79,80].

There are also some technical factors that can increase the likelihood of cybersickness occurring. These issues include noticeable lags (delays in the visual display can cause symptoms), position tracking errors (better head tracking reduces symptoms), and flicker in the visual display [71,72].

Cybersickness is one of the most uncomfortable issues that comes with VR usage, and if users continue to experience these uncomfortable symptoms, this can present a huge hindrance to the widespread development and utilization of VR applications [72,77].

### 4.2. Accessibility

As VR technology evolves, it is becoming more accessible, especially compared to its earlier stages. The cost of VR headsets on the market is still higher than most people can afford, but their current pricing is on par with most gaming consoles. Headsets such as Oculus Quest 2 cost about $300 for the base model and can be fully operated without the need for a computer, making it one of the more accessible headsets on the market. Most other headsets require using a computer that is “VR-ready”, meaning a high-end computer with a powerful graphics card that can manage VR applications. VR-ready computers tend to be more expensive than most computers, making this type of VR headset more expensive overall and out of reach for most people. This makes cost one of the larger barriers for people to get into VR as regular consumers, which is a hindrance to the growth of VR as a household technology.

VR as a field also includes augmented reality (AR) and mixed reality (XR), which are less immersive forms of virtual experiences where users still operate in the real world with a virtual overlay. AR and XR applications are more accessible to people due to their development for use on mobile devices, which are much more common with most people owning or having access to one. A common example of this type of application is AR games such as the popular *Pokémon Go*, which combines using a smartphone with a physical exploration of the real world [81] in search of “Pokémon” around them that can only be observed via their phones. Distances are tracked based on a user’s steps, and users can connect fitness apps to the game in order to increase rewards gained from crossing long distances. These types of games and applications can encourage people to be more physically active by gamifying the walking experience [82]. Similar smartphone games and applications can be a more accessible entry point for people interested in VR but who lack the funds to invest in an immersive headset and computer setup.

## 5. Conclusions

This literature review has shown how virtual reality technology has the potential to be a greatly beneficial tool in a multitude of applications and a wide variety of fields. Current applications span different domains such as engineering, education, medicine, and entertainment. With VR technology gaining popularity and traction, more VR applications can be further utilized in the future, both in improving current use cases as well as expanding to more domains. The hope is that with more VR technological breakthroughs and development, the current limitations and issues can be overcome, making long-term VR usage more realistic and accessible to more people.

Overall, VR as a technology is still in its early stages, but more people are becoming interested in it and are optimistic about seeing what kind of changes VR can make in their everyday lives. However, more and more application scenarios are under development by experts from different fields, which allows for more specific applications and development. With how rapidly modern society has adapted to personal computers and smartphones, VR has the opportunity to become the next big technological turning point that will eventually become commonplace in most households.

## Data Availability

No new data were created or analyzed in this study. Data sharing is not applicable to this article.
